# Can AIDS stigma be reduced to poverty stigma? Exploring Zimbabwean children's representations of poverty and AIDS

**DOI:** 10.1111/j.1365-2214.2011.01311.x

**Published:** 2012-09

**Authors:** C Campbell, M Skovdal, Z Mupambireyi, C Madanhire, L Robertson, C A Nyamukapa, S Gregson

**Affiliations:** * Institute of Social Psychology, London School of Economics and Political ScienceLondon, UK; † Imperial College LondonLondon, UK; ‡ Department of Health Promotion and Development, University of BergenBergen, Norway; § Biomedical Research and Training InstituteHarare, Zimbabwe

**Keywords:** Africa, AIDS, children, poverty, social representations, stigma

## Abstract

**Objective:**

We use children's drawings to investigate social stigmatization of AIDS-affected and poverty-affected children by their peers, in the light of suggestions that the stigmatization of AIDS-affected children might derive more from the poverty experienced by these children than from their association with AIDS.

**Methods:**

A qualitative study, in rural Zimbabwe, used draw-and-write techniques to elicit children's (10–12 years) representations of AIDS-affected children (*n*= 30) and poverty-affected children (*n*= 33) in 2009 and 2010 respectively.

**Results:**

Representations of children affected by AIDS and by poverty differed significantly. The main problems facing AIDS-affected children were said to be the psychosocial humiliations of AIDS stigma and children's distress about sick relatives. Contrastingly, poverty-affected children were depicted as suffering from physical and material neglect and deprivation. Children affected by AIDS were described as caregivers of parents whom illness prevented from working. This translated into admiration and respect for children's active contribution to household survival. Poverty-affected children were often portrayed as more passive victims of their guardians' inability or unwillingness to work or to prioritize their children's needs, with these children having fewer opportunities to exercise agency in response to their plight.

**Conclusions:**

The nature of children's stigmatization of their AIDS-affected peers may often be quite distinct from poverty stigma, in relation to the nature of suffering (primarily psychosocial and material respectively), the opportunities for agency offered by each affliction, and the opportunities each condition offers for affected children to earn the respect of their peers and community. We conclude that the particular nature of AIDS stigma offers greater opportunities for stigma reduction than poverty stigma.

## Introduction

Stigma and discrimination are key obstacles to the well-being of HIV/AIDS-affected children (Clay *et al*. 2003; Cluver *et al*. 2008; Skovdal *et al*. 2009). Some research explains them in terms of the links between HIV/AIDS and the anxiety-provoking or socially embarrassing issues of sexuality, physical contagion and death (Crawford 1994; Campbell *et al*. 2005; Campbell & Deacon 2006). Others have argued that the stigmatization of people with HIV/AIDS is driven more by their poverty than by their link to AIDS (Foster *et al*. 1997; Strode & Barrett-Grant 2001; Castro & Farmer 2005; Abadía-Barrero & Castro 2006; Bond 2006), arguing that stigma represents a realistic response to economically unproductive individuals, or those who threaten to drain scarce economic resources in economically challenged communities (Campbell *et al*. 2011). In relation to children, some researchers have speculated that HIV/AIDS-related stigma might often be driven more by the impoverished status of the AIDS-affected child than their association with HIV/AIDS *per se* (Foster *et al*. 1997; Strode & Barrett-Grant 2001). No empirical research has explored this issue however. To contribute to understandings of how stigma is constructed and resisted by children, and in the interests of informing anti-stigma interventions and policies, we compare and contrast children's representations of peers affected by AIDS and those affected by poverty.

The form and content of stigma varies across cultures and socio-economic contexts. Using Goffman's (1963, p. 13) characterization of stigma in terms of ‘an attribute that is significantly discrediting, which in the eyes of society, serves to reduce the person who possesses it’, we view stigma as the blend of affective, cognitive and behavioural responses to those bearing such ‘discrediting attributes’, constructed within a collectively negotiated body of social knowledge or representational field (Dovidio *et al*. 2003).

Deacon and Stephney (2007) argue that children's experience of stigma may vary according to their sexual maturity, gender, degree of poverty and local understandings of child vulnerability. They suggest poverty may provide a reason for differential treatment of orphans within a household, concluding that in many cases poverty and HIV/AIDS-related stigma should be seen in the context of orphanhood. Bond (2006) argues that HIV/AIDS-affected children may experience their life situations (e.g. not being in school, having to work to help sustain their fostering household) as stigmatizing, even if their situations are the result of poverty rather than AIDS *per se*. Furthermore, she suggests that families or communities with limited access to resources may use the language of blame and shame to refer to AIDS-affected children to justify their lack of support for them, deepening children's experience of stigma.

However, children are not only stigmatized against, they can also stigmatize. While numerous studies have explored children and young people's attitudes and responses towards *adults* with HIV/AIDS (e.g. Castle 2004; Letamo 2004; Buseh *et al*. 2006; Maughan-Brown 2006; Bhana 2008), few studies have explored children's perspectives of AIDS-affected *children* (Campbell *et al*. 2010) or their stigmatizing responses to HIV/AIDS-related peers (Cluver & Orkin 2009). To address this gap, and begin to untangle the relationship between HIV/AIDS-related stigma and the stigma of poverty, we compare children's representations of poverty-affected and AIDS-affected children.

## Conceptual framework

Social representations theory (SRT) is concerned with the representational fields, or systems of socially constructed common sense knowledge, including values, attitudes and practices, that people use to orientate themselves in their social world (Moscovici 1973). SRT provides a useful framework for exploring the meanings children co-construct to make sense of their social worlds in particular contexts. SRT is particularly useful for studying stigma because of its emphasis of the role of in-group–out-group boundaries in representational construction (Howarth 2002), and the role of unconscious anxieties in shaping the representational process (Joffe 1999). In coping with life's stresses and anxieties, social groups may collectively construct representational boundaries to distance themselves from an out-group ‘other’ onto whom they project their own fears and anxieties about life's uncertainties – such as death or sexuality or the seeming randomness of life's afflictions – characterizing this ‘other’ with stigmatizing attributions, blame and shame. While such representations lead to marginalization and suffering by the stigmatized (in this case AIDS-affected children), they can give the stigmatizors (other children living in contexts of AIDS and poverty, but perhaps not as seriously or immediately affected as some of their peers) a sense of comfort and safety through creating psychological distance between themselves and those afflicted by the life challenges they dread. It is against this background that we locate our study of children's representations of their AIDS- or poverty-affected peers, mapping out the collectively constructed representational fields within which children's responses to their peers are negotiated.

## Methodology

This study forms part of a larger project entitled ‘Studies of behavioural and STD control interventions to limit the socio-demographic impact of the HIV-1 epidemic in Zimbabwe’ and was granted ethical approval by the Medical Research Council of Zimbabwe (MRCZ/A/681) and a research committee at the London School of Economics. Written informed consent was obtained from participating children and their parents/guardians.

### Study area and sampling

Data were collected from rural and urban sites in Manicaland, eastern Zimbabwe, in 2009 and 2010. Manicaland's HIV prevalence rate is 16.57% for men (17–54 years of age) and 20.5% for women (15–44 years of age) (Gregson *et al*. 2007) with stigma cited as a key challenge to HIV/AIDS management (Duffy 2005; Sambisa *et al*. 2010). Sixty-three Shona-speaking children, aged 10 to 12, were recruited through snowball and convenience sampling. This age group, mostly not yet sexually active, has not yet developed all of the sexual anxieties experienced by sexually active adults – making them a particularly fertile age group for anti-stigma interventions – but are old enough to communicate meanings and emotions through drawing and writing. We sought a wide spectrum of representations, including children from rural and urban areas. Rural children (*n*= 32) were identified through village guides known to the research team and urban children through primary schools (*n*= 31). To compensate for their time, children were given two bars of soap.

### Data collection and analysis

The draw-and-write technique (cf. Pridmore & Bendelow 1995; Backett-Milburn & McKie 1999) provides children less comfortable with writing with an additional platform for communication (Pridmore & Bendelow 1995), and gives children space for the slow construction of narratives. Two Shona-speaking fieldworkers, qualified social workers, invited 33 children to draw a picture and write a story about a child in their community whose family was affected by poverty and 30 children to draw a picture and write a story about a child in their community whose family was affected by AIDS. In urban settings this was done in classrooms during school break time. Rural children conducted the exercise at home after school. An initial analysis of the urban and rural drawings and stories found no differences in representations, and they were pooled and analysed collectively. SRT is concerned with the diversity of representations in a social group, so we sought to map out features of a collectively constructed representational field rather than documenting attitudes conceived of as properties of individuals (Gaskell 2001). We used thematic content analysis (Attride-Stirling 2001) to analyse stories and drawings by labelling text segments or areas of the drawings, generating 63 codes which were grouped into themes (see Table 1).

**Table 1:** Representational field of poverty- and AIDS-affected children

	No. of responses (%)		
		
Representations (codes)	AIDS-affected children (*n*=30)	Poverty-affected children (*n*=33)	*P*-value
Child			
**Admiration** (good qualities, high spirit, work for social change)	7 (23%)	0	0.003
**Good looks** (beautiful girl, handsome boy)	0	16 (48%)	<0.0001
**Engagement in work/caregiving** (child-head-of-house, domestic work, migratory work, caregiving, income generation)	22 (73%)	13 (39%)	0.007
**Orphanhood** (stay with grandparent, street child, no parents)	0	19 (58%)	<0.0001
**Poverty** (poor nutrition, suffer from disease, poor housing, neglect, begging for food, dirty, patched clothing)	3 (10%)	28 (85%)	<0.0001
**Psychosocial distress** (upset, despair, lonely, worry, abuse)	13 (43%)	0	<0.0001
**Stigmatized** (fear of touching child, treated badly by peers, no friends, ostracization)	12 (40%)	0	<0.0001
Family			
**Parents engage in ‘bad’ behaviour** (drinking, promiscuity)	8 (27%)	5 (15%)	0.259
**‘Lazy’ parents** (father not bothered, blame fathers, ‘lazy’ parents)	0	8 (24%)	0.004
**Sick parents** (sleep all day, bedridden, looking messy)	22 (73%)	0	<0.0001
**Unable to work** (weak, tired, unproductive)	5 (17%)	0	0.015
**Parents engage in informal work** (informal)	0	5 (15%)	0.026
**Resources** (farm land, animals, close family ties, extended family)	17 (57%)	5 (15%)	0.001
School			
**Performs well** (studies hard, does well in school)	0	7 (21%)	0.007
**Lacks concentration** (mind is at home)	2 (7%)	0	0.132
**School absence** (dropouts, poor attendance, too busy for education)	6 (20%)	8 (24%)	0.686
**Lacks materials** (books, uniforms)	0	11 (33%)	0.001
**Unable to pay fees** (lack school fees)	0	6 (18%)	0.014
Community			
**Child accepted** (plays with friends, seen as a good child)	8 (27%)	6 (18%)	0.418
**Support** (local church, neighbours, non-governmental organizations, local organizations)	8 (27%)	5 (15%)	0.259
**Peer support** (friends help with farming, fetching water with friends)	4 (13%)	0	0.030

Frequencies in Table 1 refer to the number of children presenting a particular theme in their drawings and/or stories. If a child both drew a sick parent lying in bed and spoke about a bedridden parent, these would both be coded as ‘bedridden’. In our tally, however, we would only count the code once from that particular child. Thus, for the theme ‘sick parents’, 22 out of 30 AIDS-affected children drew and/or wrote about the sick parents of children affected by AIDS. Chi-squared tests (cf. Kirkwood & Sterne 2003) were used to compare the frequencies with which each theme was represented by AIDS-affected children and poverty-affected children, and *P*-values are presented in Table 1. The small sample of children limits our ability to draw strong conclusions from statistical tests or to adjust for possible differences between the two groups of children. However, we believed it was helpful to include this quantitative analysis to aid our interpretation of the qualitative analyses.

Below, we extract four core themes from the description of representations in Table 1 to demonstrate differences and similarities in representations of AIDS-affected or poverty-affected children. To an extent, separating children's drawings of AIDS and of poverty is a false distinction, given that poverty and AIDS will usually be intertwined in the lives of Zimbabwean children (Howard *et al*. 2006). However, it was a useful research strategy insofar as we found clear distinctions in representations when children were asked to depict each problem in isolation, suggesting that for children, each problem has its own clear and specific connotations.

## Findings

### Children's needs and difficulties

Counter-intuitively, poverty-affected children were far more likely to be represented as orphaned than AIDS-affected children (58% vs. 0% respectively, *P* < 0.0001). Poverty-affected children were often described as orphaned, living on their own, on the street or most often with their grandparents. Interestingly, given the western preoccupation with orphanhood as a lens for viewing the epidemic's impact on children, no AIDS-affected children were described as orphaned. This supports arguments that the depiction of AIDS-affected children as orphaned may be a product of the western imagination rather than reflecting the realities of AIDS-affected children, most of whom have at least one living parent, and those without often absorbed into extended families (Meintjes & Giese 2006).

Nearly all (85%) children depicting poverty-affected peers provided details of their impoverishment.

This poor family has ten children. They go to school without uniforms, shoes and bags. One of the older children is a street kid. Last year they nearly died of cholera. They eat dirty food and drink dirty water. One of the girls is mad. The house they live in is dirty and the roof has holes. (urban boy, 12, describing poverty-affected family)

Poverty-affected children were said to have poor diets, be more likely to contract infectious diseases, live in poor housing and dirty conditions and be neglected. They were frequently depicted in patched clothing. While a few children did mention the poverty of AIDS-affected children (10% vs. 85% for poverty-affected children; *P* < 0.0001) in the AIDS drawings and stories, AIDS-affected children were more often represented as suffering from psychosocial distress (43% vs. 0% for poverty-affected children; *P* < 0.0001) and stigma (40% vs. 0% for poverty-affected children; *P* < 0.0001) than poverty.

The people of the community are not giving any support to this family. They don't even go to see them because they think that they will get the virus. The children of the village run away from boy, they don't even shake hands with him. If he feels like playing with them, they start to tease him from a distance. He always gets home crying. (rural boy, 12, describing AIDS-affected boy)

Although both poverty- and AIDS-affected children were said to experience school absence (24% vs. 20% respectively; *P*= 0.686), the educational needs of children affected by AIDS and poverty were described differently, reflecting the emphasis outlined above. Poverty-affected children were depicted as lacking school fees (18% vs. 0% for AIDS-affected children; *P*= 0.014) and uniforms, books, shoes and school bags (33% vs. 0% for AID-affected children; *P*= 0.001). AIDS-affected children were said to suffer from poor concentration in school because of worries about sick parents (7% vs. 0% for poverty-affected children; *P*= 0.132).

Every day she goes to school with no school bag, no shoes, no uniform. She also does not eat lunch. (rural girl, 12, describing poverty-affected girl)

At school, he always thinks about his father; how is he doing and who may take him to the toilet. (urban girl, 11, describing AIDS-affected boy)

### Blame and sympathy for parents

Children blamed parents of both AIDS-affected and poverty-affected children. Both were said to suffer as a result of their parents' (most often fathers') ‘bad’ behaviours (27% vs. 15%; *P*= 0.259). However, the elaboration of these representations varied. Parents of AIDS-affected children were often referred to as promiscuous, highlighting the centrality of sexuality in AIDS stigma. Parents of poverty-affected children were described as too lazy to work (24% vs. 0% for AIDS-affected children; *P*= 0.004).

Her father was always drunk. He would beat her mother and go to sleep with other women. These women were prostitutes and so the father started coughing and was said to have AIDS. (urban girl, 10, describing AIDS-affected girl)

The wife and husband are both very lazy. Every morning they go to the beer hall to drink beer. They come back late at night. (urban boy, 12, describing poverty-affected girl)

**Figure 1:**
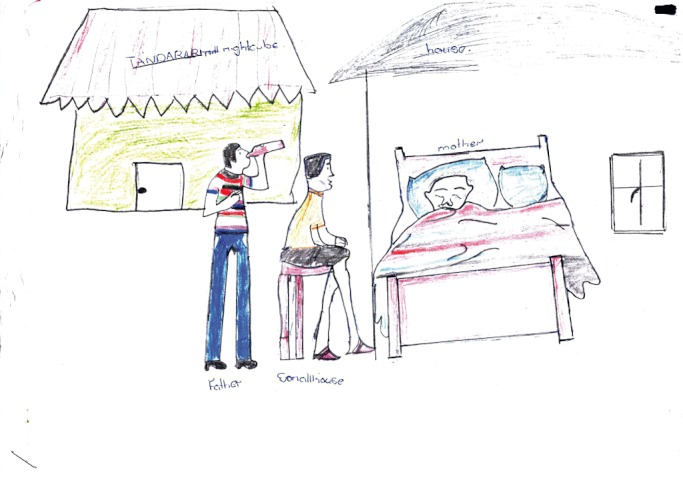
Drawing by a boy, 12, showing the father of an AIDS-affected boy outside a nightclub, watching his bedridden wife.

**Figure 2:**
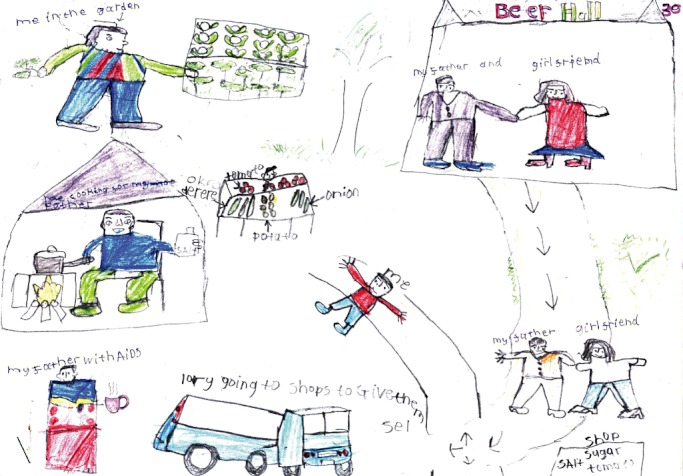
Drawing by an 11-year-old girl showing (clockwise from top right) the father of an AIDS-affected boy meeting a girlfriend in a beer hall and eventually contracting AIDS, leaving the boy to do the cooking and income generation.

Drinking alcohol and visiting beer halls, as depicted in Figs 1 and 2, were common ways children represented fathers as unsupportive and ascribed blame for the difficult circumstances facing both AIDS- and poverty-affected children.

However, there was also evidence for a degree of sympathy and understanding for parents. Five children (15%) talked about informal work activities by parents of poverty-affected children, recognizing their active efforts to survive.

The mother went to the market to sell tomatoes, bananas, apples and onions. She tries to sell these things every day. The father has no work, so he walks around every day tosell clothes and shoes. (urban girl, 12, describing poverty-affected girl)

Illustrating children's understanding of the impoverished circumstances of AIDS-affected children, 73% of children describing the lives of AIDS-affected children represented their parents as sick and bedridden (compared with 0% for those describing poverty-affected children; *P* < 0.0001), with five children (17%) saying explicitly that the parents of AIDS-affected children were unable to work.

The disease has made her mother useless, she cannot work, she spends the whole day sleeping. (urban boy, 12, describing AIDS-affected girl)

In summary, while some children did blame the parents of both poverty- and AIDS-affected children for the situation their children found themselves in (e.g. through behaviours such as drinking or extramarital sex), there was also some sympathy and understanding for their difficult circumstances, particularly for parents that were bedridden and too sick to work or those who were trying to generate income.

### Children's roles and responsibilities

Both poverty- and AIDS-affected children were represented as active contributors to household survival and livelihoods.

She had a lot of responsibilities. She had to wash all the dishes and clothes, clean the house and cook for her parents. After all of that she had to go and work for food and school fees. (urban boy, 12, describing AIDS-affected girl)

To generate income, every morning she goes to the neighbours fields to weed. (urban girl, 10, describing poverty-affected child)

However, more AIDS-affected children (73%) than poverty-affected children (39%) were represented engaging in work, domestic chores and caregiving (*P*= 0.007). In accounts of AIDS-affected children, caregiving featured more prominently, and as an added responsibility to income generation, underlining AIDS-affected children as active contributors to their household.

**Figure 3:**
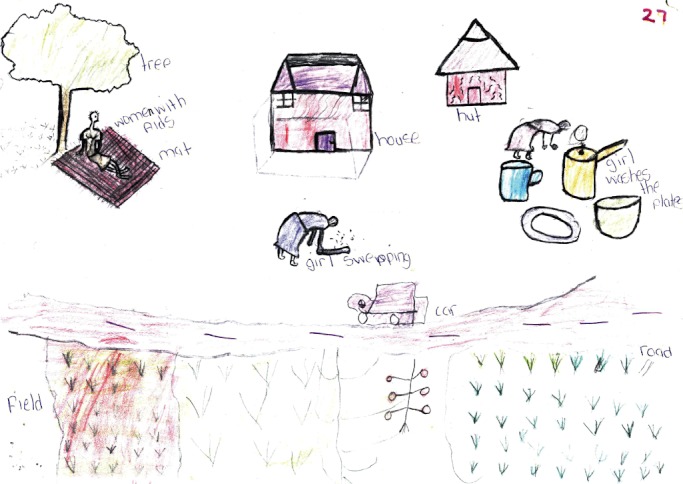
Two AIDS-affected siblings busy with domestic chores.

These two children wake up at 5am every day to help their HIV positive mother. They do work like washing the dishes, sweeping the yard and the house. They do the laundry after cleaning their mother's face and body and have carried her out of her bed on a mat under a tree. After cooking porridge they feed her. Then they wash the dishes and go to the market to buy some maize for their mother to eat in the afternoon. (urban girl, 10, describing AIDS-affected siblings) (illustrated in Fig. 3)

### Support and admiration

**Figure 4:**
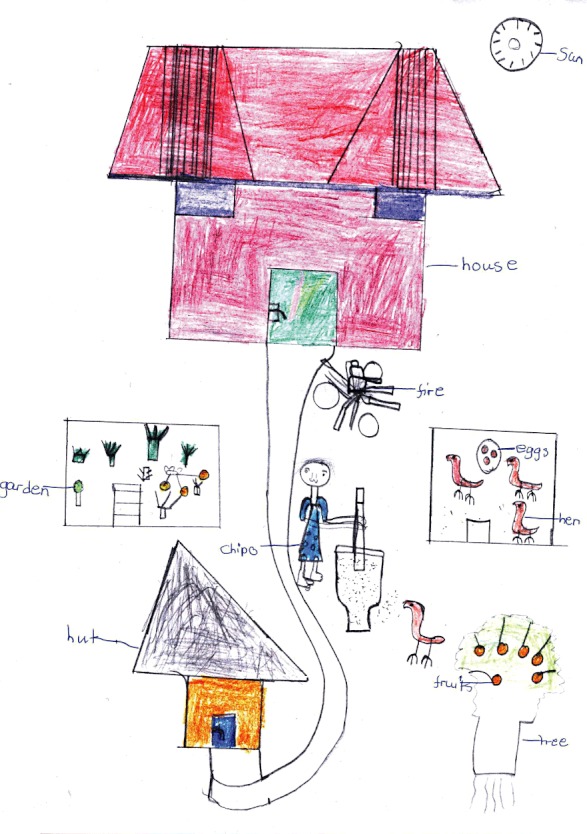
Poverty-affected child depicted as doing domestic chores, with access to fruit, meat, eggs and vegetables to sustain their livelihoods.

Children also constructed different representations of the roles and responsibilities of poverty-affected and AIDS-affected children and the resources available to them. More frequent reference was made to the local resources available to AIDS-affected children (57%) than to poverty-affected children (15%) to sustain their livelihoods (*P*= 0.001). As Figs 2–4 illustrate, farm land, livestock and poultry were some of the household-level resources children depicted.

AIDS-affected children were also very slightly more likely to be represented as receiving external support. Twenty-seven per cent of AIDS-affected children (compared with 15% for poverty-affected children; *P*= 0.259) were depicted as receiving support from neighbours, church groups and members, non-governmental organizations and local community groups.

This girl's mother is suffering from AIDS. Her neighbours care for her, they bring food for the family because she can't work. (urban boy, 11, describing AIDS-affected girl)

Every day she is late for school because she must go to the priest first to collect food. (rural boy, 12, describing poverty-affected girl)

Unlike poverty-affected children, a few children (13%) describing AIDS-affected children also spoke of the support provided by peers (*P*= 0.030).

Some of her friends help her in most of the things that her father tells her to do. (urban girl, 11, describing AIDS-affected girl)

The stories and drawings also projected a degree of acceptance and admiration of AIDS-affected children, never the case for poverty-affected children. Reflecting the many caregiving responsibilities of children living with AIDS-infected parents, 23% of participants spoke of the noble qualities of AIDS-affected children (compared with 0% for poverty-affected children; *P*= 0.003), including their compassion, hard work and good will.

She is a tolerant and compassionate girl; she is loving to her father. She is an active girl. She is faithful as a dog. She believes that one day her father will be fine. (rural boy, 11, speaking of AIDS-affected girl)

In contrast, admiration of poverty-affected children was limited to their physical appearance. In 16 stories (48%), the poverty-affected child was described as a ‘beautiful’ girl, or ‘handsome’ boy, never the case for AIDS-affected children (*P* < 0.0001). However, despite their good looks, they tended to be viewed more as passive victims of their fates, with their AIDS-affected counterparts seen as having more opportunities for agency in the face of their life challenges.

## Conclusion

This paper was inspired by previously speculative claims that the stigmatization of AIDS-affected children by their peers might relate more to the poverty associated with AIDS, than to children's association with AIDS itself. While both AIDS- and poverty-affected children were represented as battling against huge life challenges, often depicted as the result of parental shortcomings, the forms of suffering they were said to experience varied. Emphasis was laid on the physical and material deprivation of poverty-affected children (in terms, e.g. of nutrition, disease and poor housing) and their lack of supportive family networks (illustrated by their orphanhood). The suffering of AIDS-affected children tended to be described in more psychosocial terms – often in relation to distress, despair and worry about sick parents, and the anguish of their stigmatization and rejection by peers. Poverty-affected children's disturbed schooling was discussed in relation to lack of schoolbooks and uniforms, for example, as opposed to their AIDS-affected counterparts whose education was more likely to be represented as disturbed by worries and lack of concentration.

While both sets of children were depicted as active contributors to income generation, household reproduction and caregiving, such references were far more frequent in relation to AIDS-affected children. They were more likely to be represented as agents – with greater access to both livelihood resources (farm animals, land), and community support from churches, non-governmental organizations and so on, despite their cruel rejection by some peers. Poverty-affected children tended to be viewed more as passive victims of their fate, with fewer opportunities to play an active role in responding to their circumstances.

Children's representations suggest AIDS stigma cannot be reduced to poverty stigma. Each is associated with analytically distinct elements in relation to the nature of suffering (psychosocial or material), the opportunities for agency offered by each affliction, and the opportunities each condition offers for affected children to earn respect of peers and community support. As mentioned above, our findings of differences in the representations of AIDS-affected and poverty-affected children are primarily an issue of academic interest, given that, in reality, the majority of AIDS-affected children also live in poverty (Deacon & Stephney 2007). However, these academic findings do have strong implications for stigma reduction interventions in the HIV/AIDS field. These are that, given that *both* poverty and AIDS are seen as socially discredited states by children, AIDS-affected children are potentially open to social rejection from two fronts. Both of these need to be tackled – through parallel efforts to ameliorate the impacts of negative social reactions to people affected by AIDS, with material-level efforts to facilitate their livelihood opportunities.

Tackling poverty stigma may be a stronger challenge than tackling AIDS stigma, given that children's representations of AIDS-affected children contain more elements of admiration and respect for the effective roles such children play in supporting their sick parents and households. The existence of germs of respect for a stigmatized group is a powerful symbolic resource for community stigma reduction programmes. The counterposing of positive and negative representations may form the starting point for community dialogue leading to new and more positive views of a previously stigmatized out-group (Campbell *et al*. 2010). Our findings suggest that more promising symbolic resources exist for the challenge of tackling the stigmatization of AIDS-affected children than those affected by poverty.

**Key messages**

- Both poverty and AIDS are seen as socially discredited states by children.
- AIDS stigma is quite distinct from poverty stigma.
- Children affected by AIDS or poverty are perceived to have different opportunities for agency and to earn respect of their peers and the support of the community.
- As many AIDS-affected children live in deprivation, they are potentially open to social rejection from two fronts.
